# Co-Designing a Strategy for Implementing the SPARC Holistic Needs Assessment Tool in the Colombian Clinical Context

**DOI:** 10.3390/healthcare11222917

**Published:** 2023-11-07

**Authors:** Cindy V. Mendieta, Esther de Vries, Jose Andrés Calvache, Sam H. Ahmedzai, Gillian Prue, Tracey McConnell, Joanne Reid

**Affiliations:** 1 PhD Program in Clinical Epidemiology, Department of Clinical Epidemiology and Biostatistics, Faculty of Medicine, Pontificia Universidad Javeriana, Bogota 110231, Colombia; mendieta-c@javeriana.edu.co; 2 Department of Nutrition and Biochemistry, Faculty of Sciences, Pontificia Universidad Javeriana, Bogota 110231, Colombia; 3 Department of Clinical Epidemiology and Biostatistics, Pontificia Universidad Javeriana, Bogotá 110231, Colombia; estherdevries@javeriana.edu.co; 4 Department of Anesthesiology, Universidad del Cauca, Popayan 760032, Colombia; jacalvache@unicauca.edu.co; 5 Medical School, The University of Sheffield, Beech Hill Road, Sheffield S10 2RX, UK; s.ahmedzai@sheffield.ac.uk; 6 Medical Biology Centre, School of Nursing and Midwifery, Queen’s University Belfast, Belfast BT9 7BL, UK; g.prue@qub.ac.uk (G.P.); t.mcconnell@qub.ac.uk (T.M.)

**Keywords:** co-design, integrated care, palliative care, oncology

## Abstract

In Colombia, timely access to palliative care (PC) is hampered by difficulties in identifying and referring to necessary services. The SPARC (Sheffield Profile for Assessment and Referral for Care) instrument provides a holistic needs assessment to improve referrals for different forms of care. SPARC was recently validated in Colombian Spanish (SPARC-Sp) but has not yet been implemented in clinical practice. We undertook workshops that aimed to co-design an implementation strategy to inform a future trial testing SPARC-Sp in the Colombian healthcare system. Workshop attendees included patients, informal caregivers, healthcare professionals, volunteers, administrative staff and decision makers. Discussions within the workshops refined implementation and dissemination strategies for SPARC-Sp in practical scenarios. Results include the need for education, clarification and demystification of PC and the lack of time and skills of professionals to identify patients’ needs. Attendees recognized SPARC-Sp as a valuable tool for highlighting patients’ concerns, whose adaptations are needed in Colombia to address the low literacy of the population and specificities of the healthcare system. We proposed local adaptations to SPARC-Sp and produced five educational videos aimed at health professionals, patients and caregivers to strengthen understanding of holistic needs in PC while building a strategy for SPARC-Sp implementation in the Colombian context.

## 1. Introduction

Selecting appropriate strategies to implement interventions in health represents a considerable challenge: often, negative attitudes may arise toward the new intervention, and a change in habits is notoriously difficult. Implementation strategies are methods or techniques used to enhance the adoption and implementation of interventions. One way of designing an implementation strategy is through co-design: involving stakeholders in patient/personal and public involvement (PPI) events together with the research team to co-design processes for use in future real-world clinical practice. Co-design is intended to be a social, participative and democratic process and is very suitable to promote the participation of patients, caregivers and healthcare professionals. It is based on the premise that it empowers all these stakeholders to become active players in the development of services that they will use in the future [1]. It is noted to be a useful approach to encourage collaborative working across patients, caregivers and healthcare professionals in complex healthcare environments [2,3]. Co-design methods have been used to achieve more contextually adapted and adjusted strategies [4,5,6,7] to increase the likelihood of successful implementation of interventions in healthcare [8]. Accounting for such stakeholder priorities in co-design processes provides a better understanding of the perceptions, needs, barriers and other practical issues that can optimize the implementation.

The provision of palliative care (PC) in Colombia is regulated by Law 1733 of 2014. However, multiple barriers for patients in need of PC have been described, related to the centralization of PC services in larger cities, the limited offer of home-based PC services, fears of PC based on myths and the lack of awareness in the community of the existence of PC [9,10,11]. Patients are often referred late, and physicians indicate having difficulties identifying holistic care needs and referring to PC in a timely manner [9,10,11]. There is currently no validated comprehensive needs assessment tool for palliative care in use.

Recently, we translated and culturally adapted the Sheffield Profile for Assessment and Referral for Care questionnaire to Colombian Spanish (SPARC-Sp) (unpublished information). The SPARC (Sheffield Profile for Assessment and Referral for Care questionnaire to Colombian Spanish) assesses patients’ holistic needs [12] and is a generic tool, not restricted to any specific disease. It has 56 items included within eight domains: communication and information issues, physical symptoms, psychological issues, religious and spiritual issues, independence and activity, family and social issues, treatment issues and personal issues [12]. SPARC was initially used in the United Kingdom (UK) [12] but is now also used in Poland [13], Korea [14] and Taiwan [15]. SPARC was originally produced by a process of co-design between healthcare professionals, patients, informal caregivers and consumer groups [4,8,12]. The English language version of SPARC is available in the annex of the paper by Hughes et al. [12].

Mechanisms to assist healthcare professionals and the Colombian healthcare system to meet the increased PC need for patients with cancer have been called for [16], and the results of the SPARC-Sp validation were promising, but some further local adaptations were suggested. Clinicians and patients involved in the evaluation process were very positive about SPARC-Sp, and the team decided that it would be useful to study the implementation of SPARC-Sp in clinical practice. However, introducing any modifications into clinical practice presents certain difficulties [17]: the novelty of such an instrument in the Colombian clinical setting; the misconceptions that exist surrounding holistic care and PC in Colombia [18,19]; the wide variety of geographical, social, political and cultural contexts within Colombia; and the lack of integration of PC [15]. Thus, in order to test the implementation of SPARC-Sp in Colombia robustly, it is crucial to first acknowledge and address the local social and healthcare dynamics of the country. To do so, the collaborative design of an implementation strategy is viewed as an essential phase. The aim of this project was therefore to co-design an implementation strategy that would later facilitate and inform a future trial testing SPARC-Sp in the Colombian healthcare system. Accordingly, we invited key stakeholders consisting of patients, informal caregivers, healthcare professionals, health administrative staff and financial staff from various regions of Colombia (urban and rural and from different geographical areas) to participate in a co-design implementation strategy to inform future research, allowing testing of SPARC-Sp in the Colombian real-world clinical settings.

## 2. Methods

**Figure 1:**
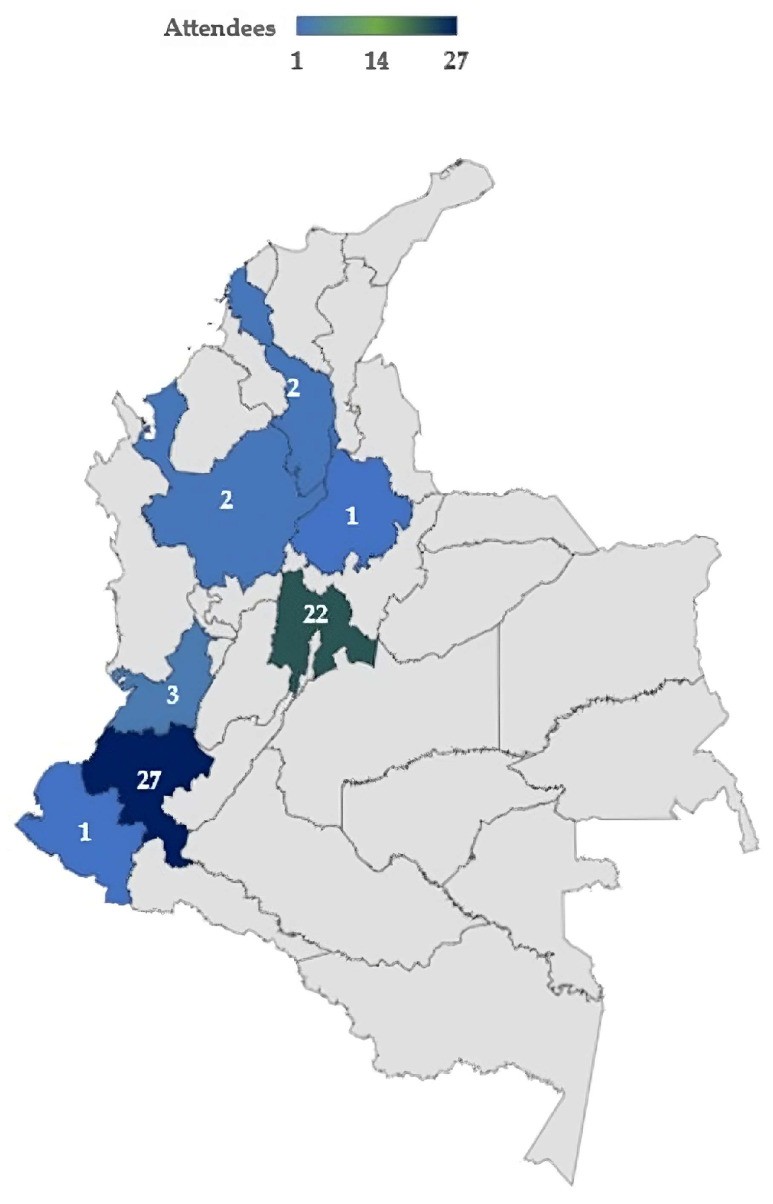
Geographical spread of attendees.

This project used qualitative workshops (n = 4). As we needed to explore several aspects of a potential implementation strategy, from the point of view of professionals, patients and caregivers, a series of meetings was necessary—every time continuing the process. This, as well as the depth of the data required, indicated the need for a qualitative co-design methodology. This qualitative methodology does not aim for a representative sample. Rather, attendees are purposively invited (based on their personal or professional experiences, as outlined in Section 2.1) as key informants based on their ability to contribute to discussions on the topic of the workshops [20]. We outlined the geographical spread of attendees within the workshops (Figure 1), so it is clear what groups are not represented. Furthermore, within the limitations (Section 5), we highlighted key demographics of the wider population who are not represented and the need for additional work in this area.

### 2.1. Workshop Attendees

Within this project, we undertook four co-design workshops. We purposively invited attendees for the co-design workshops who were key informants based on their ability to contribute to discussions on the topic of the workshops and aim of the project, the appropriateness of which is outlined in the literature. Workshop attendees (Figure 1) were contacted and invited through personal contacts and social networks (invitation on Facebook page and Twitter of “Proyecto colibrí”, email and Whatsapp) of the Colombian co-authors (CVM, EdV, JC) and a patient group from a Colombian University. We sent an email invitation addressed to patients, caregivers and healthcare professionals to participate in the workshops. They were also asked to resend the invitation to people in their network who might be interested (snowballing). Patients with chronic diseases and persons who were or had been either professionally or personally involved in the care of persons with chronic and potentially life-threatening illnesses were invited to participate in at least one of the workshops; those who accepted were asked to identify other potentialattendees in their circle of acquaintances (snowballing). This way, workshop attendees represented a multidisciplinary group that comprised patients with chronic non-communicable diseases (including cancer), family members, informal (non-paid) caregivers, health administrative staff, financial staff and healthcare professionals (physicians, medical students, nurses, dentists, physiotherapists, nutritionists, occupational therapists, speech and language therapists), coming from urban (Bogota, Medellin, Bucaramanga, Cali) and rural areas (Popayan, Pasto) of Colombia (Figure 1).

### 2.2. Organization of the Co-Design Workshops (November 2022 to July 2023)

The co-design process began with two virtual workshops (using Microsoft Teams Classic 1.6.00.11156). The total number of workshops was not predefined, as we relied on the suggestions from the attendees to determine the main facilitators and barriers and from there to move forward [21]. In the end, the two groups of attendees (healthcare professionals and patients/caregivers) each attended four workshops (a total of 8 workshops were organized: for details, see Table 1). Each online (remote) workshop was recorded and transcribed, and after each workshop, the main discussion points were summarized and presented as the basis for the next workshop. These brief presentations were pre-recorded and subtitled (those presented in English subtitled to Spanish, and vice versa) to facilitate understanding and communication between the UK- and Colombia-based groups. Four workshops were organized in virtual mode via Microsoft Teams and four face-to-face. Two of the face-to-face workshops were organized in Bogota (the capital city of Colombia) and two in Popayan, a small city in a rural area of Colombia. We divided the attendees in these face-to-face workshops into (a) healthcare professionals, administrative and financial staff and (b) patients and informal caregivers.

Each workshop lasted for approximately 90 min. The first co-design workshop sought to identify barriers and facilitators to improving access to PC and the dissemination of SPARC-Sp in the Colombian healthcare context. The first workshop began with a short introduction to the co-design team organizers (JR, EdV, SHA, JAC, GP, TM, CVM), followed by a brief explanation of the concept of holistic needs and why it is important to measure them, the potential use of the SPARC-Sp instrument for this measurement and the experience of its translation and validation in Colombia.

In addition, we introduced the concept of co-designing implementation strategies and the importance of identifying barriers and facilitators to improve access to PC in Colombia. Each presentation was pre-recorded on video and, when presented in English, subtitled in Spanish, and vice versa. At the end of each presentation, we discussed the contents with the attendees: did they understand the concept and agree or not on the importance; what would they consider important; and what barriers did they perceive or expect when communicating about or measuring holistic needs?

In the second workshop, we summarized the discussion points of the first workshop on SPARC-Sp implementation barriers and facilitators. These first two workshops had a mixed group of attendees (patients, informal caregivers and healthcare professionals together). Workshop three was divided into four sub-workshops (3a–3d) conducted as face-to-face meetings for patients and informal caregivers (3a,3c) separated from those for professionals (3b,3d), organized in Bogota and Popayan. During these face-to-face workshops, we conducted two exercises, gaining feedback on a series of videos and the SPARC-Sp tool. The video scripts were prepared by the team and recorded again by SHA, subtitled in Spanish and presented at the face-to-face workshops in Bogota and Popayan. We introduced the paper version of the SPARC-Sp instrument among the attendees and guided the discussion with questions proposed by the team. Attendees could write or audio-record their opinions about SPARC-Sp.

Workshop four was conducted remotely and divided into two sub-workshops: one for healthcare professionals (4a) and one for patients and informal caregivers (4b). During the third (3a–3d) workshop, we shared an educational proposal for disseminating SPARC-Sp in rural and urban areas of Colombia. During the fourth (4a–4b) workshop, we presented the revised educational proposal based on the feedback received during the face-to-face workshops. All workshops had an iterative approach in which we checked with the attendees whether their perspectives were correctly represented. In case of low attendance, we sent workshop materials (e.g., videos) via email and asked for the attendees’ feedback.

### 2.3. Analysis

One of the researchers (JR) conducted the thematic analysis [22], which was carried out across 6 steps. Step 1: Becoming familiar with the data. Within this step, the recorded discussion from each workshop was transcribed and translated (from Spanish to English) by two bilingual researchers (CVM and EdV). Then, JR read and re-read it. Step 2: Generating codes. Within the transcripts, any statement that appeared to provide insight into an implementation strategy to inform future research, allowing testing of SPARC-Sp in Colombian clinical settings, was coded. The codes helped to organize the data in a way that related specifically to the aim of this project. Step 3: Generating themes. Within this step, initial themes from the codes within the transcripts were brought together. Themes aimed to capture as fully as possible attendees’ views on what would be required to inform an implementation strategy for future research, allowing testing of SPARC-Sp in Colombian clinical settings. Themes were then discussed with all authors prior to each co-design workshop, and the authors could modify or suggest additional items or categories. These consolidated findings were again translated into the native language of the attendees (Spanish, by CVM and EdV) and presented at each workshop, for evaluation by the attendees. Step 4: Reviewing themes. In line with this step, themes generated were checked to ensure they accurately reflected the data set as a whole, across each of the workshop transcripts. This involved reading all transcript sections associated with each theme to ensure they supported it and that the themes were coherent. Step 5: Defining and naming themes. Within this step, final themes (n = 3) were confirmed and named (Table 2). The aim of this step was to *“identify the ‘essence’ of what each theme is about’* ([21], p.92) and ensure that was accurately conveyed in the theme titles used. Step 6: Locating examples. In line with step 6, we selected direct quotations from the workshop discussions to help exemplify the three themes generated from analysis (as presented within Section 3).

### 2.4. Ethical Considerations

The co-design workshops were designed as PPI events and discussed with all involved institutions. Research governance in Queen’s University Belfast, Pontificia Universidad Javeriana and Universidad del Cauca institutional guidance on the appropriate use of PPI was adhered to. As such, ethical approval was not required for the PPI co-design workshops. Attendees gave their permission to record, transcribe and use their information for each of the workshops and future publications. The transcripts of the workshops were anonymized to protect the identity of the attendees. Attendance at the workshops was voluntary, so attendees were free to leave the workshop at any time. This also explains the varying number of attendees at each session. We provided financial compensation in the form of a voucher of approximately 12 USD to those who attended the face-to-face workshops to compensate for the time and travel costs of the attendees to the venue.

## 3. Results

The order, mode (remote or face-to-face), topic, methods, type and number of attendees in each workshop are provided in Table 1. As the content of each subsequent workshop depended on the results of the previous one, the main discussion points of the different workshops are organized below in chronological order together with a brief description of the workshop methodology. Each attendee could be active in a maximum of four workshops. The first two workshops had a mixed composition of service providers and service users. However, to encourage participation and discussion among attendees and avoid shyness among patients and caregivers in the presence of physicians and other healthcare workers, when it came to the face-to-face workshops and subsequent feedback, we decided to divide the population into healthcare professionals and patients or informal caregivers, resulting in a total of eight workshops. Thematic analysis of discussions within the workshops resulted in three themes being identified (Table 2). Each of these will be discussed in turn in the subsequent sections.

### 3.1. Determination of Barriers and Facilitators in Access to Palliative Care and SPARC-Sp Implementation

The main objective of the first and second workshops was to explain the importance of undertaking holistic needs assessment, present the SPARC-Sp tool, explain the concept of co-design and the methodology proposed for our series of co-design activities and facilitate a discussion on the concept of holistic care, SPARC-Sp and how to implement SPARC-Sp in the clinical setting in Colombia.

While all attendees recognized the need for an instrument (or a tool) to facilitate effective communication related to needs, several barriers were also identified. All attendees from more rural areas and some from the urban areas mentioned the low reading skills of a substantial proportion of the Colombian population as a barrier to its implementation as a self-report instrument [23]. SPARC-Sp consists of 56 questions, most of which have four response options. The substantial text, combined with some novel health-related concepts, was considered a barrier by many, despite an initial validation of the English to Spanish translation.

In relation to the identification of patients’ holistic needs, either during normal consultations or using SPARC-Sp, all attendees mentioned the limited time of professionals to identify holistic needs as a barrier: in an encounter of a few minutes, the physicians usually focused on the most common physical concerns and side effects of treatment.

“*What we specialists do is to deal acutely with the PC scenarios, to try to solve the acute situations, but very rarely do we have the time to do as holistic an approach as we would like*”.

Popayan, physician, workshop 3.

Patients and informal caregivers mentioned that they often did not feel empowered or had the time to bring up other topics during consultation. This was also considered a barrier to the effective use of SPARC-Sp: having to read or explain the questions could be considered too much of a burden for clinicians which would present a barrier to the effective use of SPARC-Sp.

“*Regarding the level of education of the patients, since not all of them can read, sometimes the option would be to have to read (the SPARC instrument) to the patient*”.

Popayan, medical student, workshop 1.

Another barrier to the optimal implementation of SPARC-Sp was related to the many misconceptions that surround PC for healthcare professionals and patients alike: many do not know what PC means and associate it with abandonment by the healthcare system, end-of-life care and suffering.

“*There is resistance to certain specialties (…). For them to refer to PC is complex (…) These specialties resist PC, they try to do everything possible, because they do not consider this [an] accompaniment of the good death*”.

Bogota, nurse, workshop 1.

In addition, attendees described financial and administrative barriers that limit availability, referral and access to PC services, especially in rural areas.

“*I can tell my patient let’s fight for that right (access to PC), even with an action of protection (tutela: legal mechanism to appeal to denial of certain treatments in the Colombian healthcare system), right of petition. It is not a favor; it is something that is already determined*”.

Bogota, physician, workshop 3.

### 3.2. Opportunities to Improve the Access to Palliative Care and SPARC-Sp Implementation

Workshop attendees also identified opportunities for action based on the identified barriers. Patients recognized the importance of the empowerment of the different actors brought about by the co-design workshops. This discussion gave attendees a sense of ownership of the implementation of PC in Colombia. All noted that PC is not only the work of the medical and nursing team but that allied and social healthcare professionals should also be involved in its implementation.

“*I work with military forces. I am in the interdisciplinary group of PC. But I have never been called (to the medical consultation of PC)*”.

Bogota, registered dietitian nutritionist, workshop 3.

Overall, attendees felt that SPARC-Sp was a useful instrument to start a conversation about holistic needs in PC. Although its application can be difficult, utilizing patient champions or volunteers could contribute to reading and answering SPARC-Sp. During the second workshop, a summary of identified barriers, facilitators and other discussion points from workshop 1 was presented, and attendees confirmed that these represented the issues raised correctly. Additionally, the original developer of SPARC (SHA) gave a series of introductory presentations on PC, communication skills and more details on the development and use of the SPARC instrument. Considering the feedback, the team proposed to consider an online version of SPARC-Sp including audiotapes of each question. Such an online version of the instrument would also allow the automated production of a summary of the identified needs, which could easily be printed and handed over or even sent electronically to the healthcare professionals prior to or during consultation, to help address the barrier of limited time. Healthcare professionals as well as patients and informal caregivers agreed that it would be important to have a complete, easy-to-understand source of information regarding PC, holistic care needs and SPARC, and it was decided to build on the contents of the messages provided in this first series of videos by SHA. The idea of making the online version with a summary of needs was welcomed by all attendees.

### 3.3. Co-Designing the Implementation Strategy for SPARC-Sp

All attendees from the four workshops agreed on the usefulness of the videos but highlighted the need to have separate videos for healthcare professionals and patients and caregivers. They also requested that the videos should be presented in Spanish rather than English, by local persons who are knowledgeable on the topic. Attendees also stressed the importance of using language and terms that would be understood by those with low literacy levels.

Upon looking at the SPARC-Sp instrument itself, attendees almost unanimously identified the need to include questions regarding how to access help to navigate the Colombian healthcare system. In addition, some attendees with low literacy levels highlighted having difficulties understanding certain specific terms (bowel problems, symptoms that are not controlled, appearance, anxiety, confusion, concentration, financial issues). Some also reported difficulties in distinguishing the four answer categories within SPARC-Sp (not at all, a little bit, quite a bit, very much) in the patient and caregiver group. Attendees also highlighted that they were positively surprised that SPARC-Sp included some questions on important issues (sexual life, thoughts about ending it all) which are not normally mentioned by healthcare professionals but which were recognized as important.

Integrating these observations and searching for a solution, it was proposed to produce new healthcare professional videos (three), narrated by members of our team: a pain and PC specialist (JAC) and two videos for patients and caregivers, narrated by a member of our team: a dietician and research assistant (CVM). In relation to the items of SPARC-Sp that attendees advised were difficult to understand, the language used was slightly reformulated. For example, colloquial ways of referring to certain ailments were added to the more formal terms to enhance understanding. In relation to the difficulties that attendees discussed in choosing between answer options, the team proposed to make a color-coded response option, following the traffic light system. This was green meaning having no needs regarding a specific issue (not at all), through light and more intense orange for increasing needs, to red being a very high need on a specific item (very much). This color coding also facilitated choosing the answer options for those attendees preferring the audio version of SPARC.

**Figure 2:**
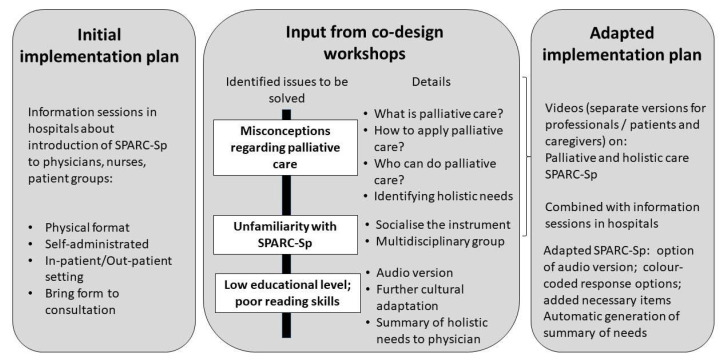
Synthesis of findings from the co-design workshops.

Based on these discussions at the face-to-face workshops, we proceeded to design and produce a series of five professional videos in Spanish [24]. These address the main needs and barriers identified during the co-design workshops, such as definitions and explanation of PC, actors involved in the provision of PC and the application of the SPARC-Sp tool. Three videos were aimed at health professionals (one on what PC is, one on who provides PC and the importance of evaluating holistic care needs and one on the SPARC-Sp tool in particular). A further two videos were produced for patients and caregivers of patients with palliative and holistic care needs (one on what PC is and one on the SPARC-Sp instrument and what it is used for) [24]. After the videos were developed, they were shared with the attendees in a separate workshop (4a–4b) for professionals and patients/informal caregivers, together with the final proposal for adaptations to SPARC-Sp and a draft plan on how to implement SPARC-Sp for future testing within a trial. This plan consisted of the following: patients, caregivers and professionals will receive the videos; the adapted online version of SPARC-Sp (with audio option and traffic light color coding of responses), which will be sent prior to consultation to be completed by the patients; the professional will receive a summary of identified holistic care needs; professionals will decide the course of action based on the consultation combined with the SPARC-Sp summary received. Figure 2 summarizes the main findings of the co-design process.

## 4. Discussion

In a total of four workshop sessions per group, with attendees from urban and rural Colombia and consisting of healthcare professionals, patients and informal caregivers, we co-designed an implementation strategy for SPARC-Sp, to inform future research that will test SPARC-Sp in clinical practice. Previous to the co-design workshops, the proposal was to inform local healthcare personnel, patients and caregivers regarding the objectives of SPARC-Sp and the intention to design, together with the attendees, a strategy for a smooth implementation of SPARC-Sp in Colombian clinical practice. As a result of the co-design process, we identified important prerequisites or adaptations necessary prior to implementing SPARC-Sp in practice: (1) educating professionals, patients and caregivers on what PC is, who provides it and why measuring holistic care needs is (a) useful and (b) how it can be done; (2) the limited literacy of a substantial proportion of the Colombian patient population, making the use of self-completed questionnaires difficult; and (3) the limited time and skills of healthcare professionals to identify and address holistic needs in PC (Figure 2). As a next step, we worked on each of these prerequisites: to address the first issue of education, we co-designed videos aimed at either healthcare professionals or patients and caregivers. The second issue of literacy was addressed by identifying other options for the application of a health instrument as an audio-based tool, for which steps are currently underway. The third issue of healthcare professionals’ limited time was addressed through the provision of a summary of identified needs to the professionals combined with the educational video regarding how to interpret and react to identified needs.

In addition, as with the first co-design workshop, we also identified the existence of many misconceptions about PC: many specialists seem to consider referral to a PC service as a professional failure; patients and caregivers fear that they will be abandoned by their doctors and the system in general when they are in PC, which can be associated with dying and feelings of despair [11,18,19]. For this reason, we decided to also work on ways to educate professionals, patients and caregivers on what palliative care and holistic care needs are and the importance of implementing PC as early as possible.

The importance of recognizing that holistic needs in PC go far beyond pain and physical symptom control is reflected by the reactions of some patient and caregiver attendees to the items addressed in SPARC-Sp, such as having a dry mouth but also sexual life and depression. Attendees were surprised that these symptoms, which they had experienced, were something that could or should be mentioned to their healthcare professionals. Neither the patients nor the professionals seemed to be aware of the need to communicate or enquire about such issues. Without such communication, it is difficult for professionals to refer to adequate supportive care professionals, and timely management of such symptoms is hampered [25]. In the literature, multiple benefits of timely and well-oriented PC are described for the patients and their families, such as better symptom control, satisfaction with treatment, reduced treatment toxicity, fewer side effects, less treatment interruption, reduced psychological stress, increased survival, improved quality of life and active involvement of the patient and family in care [18].

Currently, within the Colombian healthcare system, no validated holistic needs assessment tool for PC exists—SPARC-Sp is close to finalizing such a process, with promising results (submitted). The implementation of health instruments requires recognition of the need to address linguistic, social and cultural differences [12]. Thus, co-designing an implementation strategy to ensure it will meet the needs of the local context was seen as an essential step in this program of work to optimize the implementation and subsequent evaluation of SPARC-Sp into clinical practice. To exemplify this point, planning for the implementation of SPARC-Sp within a population that has, to an important extent, low educational levels and low literacy skills was not addressed before in the previous adaptation and implementation studies of SPARC-Sp conducted in the UK, Poland, South Korea and Taiwan, probably because of uniformly higher levels of literacy in these countries [13,14,15,26]. The initial translation and cultural adaptation to Colombian Spanish did consider using more easy-to-understand concepts. Even though during the translation and cultural adaptation process, some specific words were considered adequate by the translation panel, they were later identified as difficult to understand for some of the attendees in our co-design workshops. This highlights the large educational differences that exist in Colombia and the importance of validating such translations and adaptations to low-literacy populations—which comes with several challenges from the design to the implementation of assessment questionnaires [26].

The limited time of physicians and other healthcare professionals to identify and address holistic care needs is not unique to Colombia and represents an important challenge to numerous healthcare issues. Surbakti et al. conducted a systematic literature review in six countries (Belgium, Switzerland, Germany, Spain, Netherlands, UK), which found that the average time for a medical consultation is between 6.9 and 12.4 min [27]. Although consultation time will always be a constraint, effective communication regarding patient needs is associated with higher patient satisfaction irrespective of the length of the consultation [27]. It is hoped that self-completed assessment tools can help in identifying, summarizing and communicating needs in a more time-efficient manner. In addition, the use of SPARC among recently diagnosed thoracic cancer patients when used by hospital departments can help in identifying the levels of need that services must be able to respond to [28].

To our knowledge, there is no previous work on holistic needs assessment tools using co-design methods and PPI engagement which involve the heterogeneous populations of professionals, patients and caregivers in Latin America. It was already known that co-design can be beneficial, not only producing materials with increased relevance and acceptability for end users but also resulting in a sense of support and enthusiasm for the intervention [29].

Co-design is a promising strategy for bridging professional practice and design and implementation in community settings. This type of design has taken off in Colombia to develop a participatory, critical design that gives a voice to vulnerable or underrepresented populations [30]. Unsuccessful implementation is more likely when strategies are chosen routinely or by habit rather than purposefully addressing specific barriers with end users [31]. Within the Colombian healthcare context, the existence of unmet PC needs has been reported in the literature, and within everyday clinical practice, it may not always be identified [32]. As a first step to address this unmet need, healthcare professionals must be able to identify patients’ holistic needs through a systematized assessment using validated instruments. Additionally, equitable access to PC is advocated within the Colombian healthcare context [11]. The use of a tool such as SPARC-Sp can help to reduce disparity and achieve equity through standardized assessment and identification of such needs. If successfully implemented within Colombia, SPARC-Sp could act as an exemplar for transferability (while incorporating the country-specific cultural context) to other Spanish-speaking countries within Latin America. In summary, the implementation of co-design confers multiple advantages such as gaining different perspectives, prioritizing end users’ needs, improving knowledge of patients’ needs, providing validation of ideas, reducing strategy development time and obtaining immediate feedback, making the decision-making process more efficient [33]. In this particular endeavor, we had an additional challenge of facilitating communication with the English-speaking members of the project team and the Colombian professionals, patients and caregivers, who often do not have bilingual capacities. The use of subtitles in the videos and the presence of continuous translating (by the bilingual Colombian members of the research team) were challenging but worked well.

Communication between patients and health professionals can be challenging, with previous research suggesting that patients often perceive their perspective to be dismissed, devalued and considered less relevant than the staff-centered perspective [34]. We observed that separating the groups of patients/caregivers and health professionals made it possible to improve workshop discussions, making sure patients and caregivers felt safe and empowered to make their observations and honor these observations (Table 1). However, having a few combined workshops was useful, particularly for the healthcare professionals to hear patient views first-hand. For this to work, it is important to ensure that some vocal and very empowered patients and caregiver representatives are participating, so that once they begin to express their views, some other, less vocal members of this group will follow.

## 5. Limitations

Our work has multiple limitations. The qualitative nature of this project did not require sampling strategies that guarantee populational representativeness but rather required equal involvement of all stakeholders from health providers to end users. Although we managed to have participation from all these groups, it is possible that in some institutions, other considerations not highlighted in our workshops may have been mentioned [20,35]. We did not ask for the ethnicity of the attendees, so it is possible that indigenous populations were underrepresented. Unfortunately, we had difficulties in successfully recruiting patients and informal caregivers, resulting in a rather small group of these important stakeholders. This difficulty has to do with the lack of organization of patient groups in Colombia, the limited experience and inclusion of this population in service development and the hesitation of, in particular, lower-educated patients and caregivers in voicing their opinions in these kinds of settings. The face-to-face workshops were held in only two locations (Popayan and Bogota), limiting the face-to-face interaction with attendees from more remote areas. However, during the online workshops, we had attendees from most regions of Colombia (Figure 1), and we included a multidisciplinary group to have a broad and heterogeneous perspective, including patients and caregivers with low literacy. However, it is possible that the perspectives of regions that did not participate were not represented. Our attendees had full autonomy to influence the process and the outcome (including the number of sessions and discussion topics). We feel that, with all these efforts, we honored the premises of co-design: to ensure a high degree of user involvement and move decisional authority from the development team to and with the attendees [36]. In future research, we plan to test and evaluate the effects of the implementation of SPARC-Sp as an intervention in routine healthcare. It is envisaged that SPARC-Sp will help to facilitate the identification and communication of holistic needs with healthcare professionals and thereby serve as input for future care plans.

## 6. Conclusions

This project aimed to co-design an implementation strategy to inform a future trial testing SPARC-Sp in the Colombian healthcare system. The co-design workshops provided a contextual framework for the application of SPARC-Sp where we identified the importance of sense checking the instrument with the target population, modifying its application considering the low literacy of the population and providing a summary of needs to the physicians considering the time constraints of physicians. To address these objectives, we co-designed five videos aimed at patients, caregivers and health professionals to provide education about PC in Colombia and SPARC-Sp. The adaptations to SPARC-Sp including an audio version and the generation of a summary of needs are pending and will hopefully help to optimize implementation.

## Figures and Tables

**Table 1:** Scheduling of the co-design workshops.

Workshop Number	1	2	3a	3b	3c	3d	4a	4b
**Modality**	**Remote**		**Face-to-face Bogota**		**Face-to-face Popayan**		**Remote**	
**Purpose**	Introduction to the project. Stakeholder discussion on barriers and facilitators in relation to improving access to palliative care and SPARC-Sp in the Colombian context.	Feedback on barriers and facilitators in relation to SPARC-Sp in the Colombian context	Educational proposal for disseminating SPARC-Sp in rural and urban areas of Colombia				Presentation of the educational proposal based on the feedback received during the face-to-face workshops	
**Number of Healthcare professionals**	n:23	n:27		n:10		n:20	n:18	
**Number of Patients and caregivers**	n:7	n:6	n:16		n:17			n:2

**Table 2:** Themes from thematic analysis.

Workshop	Themes
1/2	Determination of barriers and facilitators in access to palliative care and SPARC-Sp implementation
3	Opportunities to improve access to palliative care and SPARC-Sp implementation, discussion of details of the videos
4	Co-designing the implementation strategy for SPARC-Sp

## Data Availability

The co-designed videos are open-access and are available on the Colibri Project website.
